# MR spectroscopic imaging and its association with EEG, CSF, and psychometric/neuropsychological findings in patients with suspected autoimmune psychosis spectrum syndromes

**DOI:** 10.1017/neu.2025.10036

**Published:** 2025-09-17

**Authors:** Dominique Endres, Isabelle Matteit, Katharina von Zedtwitz, Bernd Feige, Andrea Schlump, Marco Reisert, Kathrin Nickel, Kimon Runge, Katharina Domschke, Evgeniy Perlov, Alexander Rau, Harald Prüss, Thomas Lange, Ludger Tebartz van Elst, Simon J. Maier

**Affiliations:** 1 Department of Psychiatry and Psychotherapy, Medical Center - University of Freiburg, Faculty of Medicine, University of Freiburg, Freiburg, Germany; 2 Division of Medical Physics, Department of Diagnostic and Interventional Radiology, Medical Center - University of Freiburg, Faculty of Medicine, University of Freiburg, Freiburg, Germany; 3 Department of Stereotactic and Functional Neurosurgery, Faculty of Medicine, Medical Center - University of Freiburg, University of Freiburg, Freiburg, Germany; 4 German Center for Mental Health (DZPG), Berlin, Germany; 5 Luzerner Psychiatrie, Hospital St. Urban, Switzerland; 6 Department of Neuroradiology, Medical Center - University of Freiburg, Faculty of Medicine, University of Freiburg, Freiburg, Germany; 7 Department of Neurology and Experimental Neurology, Charité - Universitätsmedizin Berlin, Berlin, Germany; 8 German Center for Neurodegenerative Diseases (DZNE) Berlin, Berlin, Germany

**Keywords:** Autoantibody, neuroinflammation, MRS, neurochemistry, brain

## Abstract

**Introduction:**

Autoimmune psychosis (AP) and other autoimmune psychiatric syndromes (APS) are associated with central nervous system antibodies. This study investigated related magnetic resonance spectroscopic imaging (MRSI) signatures and their correlations with electroencephalography (EEG), cerebrospinal fluid (CSF), and psychometric/neuropsychological measures.

**Methods:**

Twenty-eight adults with suspected antibody-positive AP spectrum syndromes were compared with 28 matched healthy controls. Inclusion in the patient group was based on the APS concept, resulting in a heterogeneous group with uniform autoimmunity. MRSI was performed using a spiral-encoded Mescher-Garwood localised adiabatic selective refocusing 3D-MRSI sequence. Glutamate+glutamine (Glx), gamma-aminobutyric acid (GABA), total N-acetylaspartate (tNAA), and total creatine (tCr) were reported as ratios to tNAA and/or tCr. EEG was analysed for intermittent rhythmic delta/theta activity (IRDA/IRTA) using independent component analysis.

**Results:**

No significant differences in Glx, GABA, tNAA, or tCr ratios were observed between patients and controls. Correlation analyses in patients showed a trend for a negative association of the IRDA/IRTA rate before hyperventilation with the GABA/tCr ratio in both hippocampi and with the GABA/tNAA ratio in the left hippocampus and Glx/tCr ratio in the right putamen and pallidum. Significant positive correlations were observed between inflammatory CSF markers (white blood cell count and IgG Index) and GABA/tCr and GABA/tNAA ratios in the left caudate nucleus and right isthmus cingulate and thalamus, as well as between negative symptoms in PANSS and higher GABA/tCr ratios in the right putamen.

**Discussion:**

No group differences were identified; however, correlations suggest a link between neuroinflammatory CSF markers and negative symptoms with GABAergic signalling in patients. Multimodal diagnostic approaches may provide a better understanding of the link between neuroinflammation, neurochemistry, and EEG slowing.

## Graphical abstract

**Figure:**
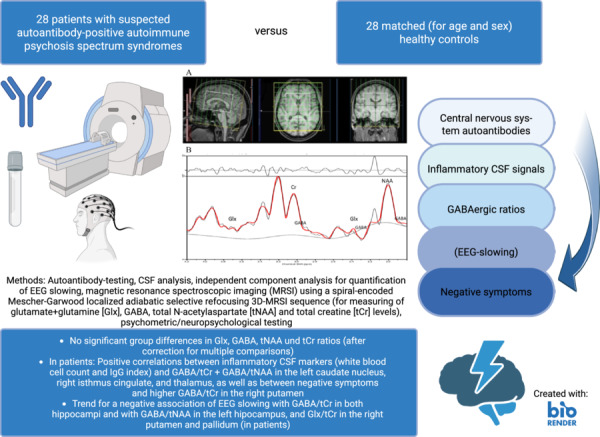

## Significant outcomes

- No significant differences in Glx, GABA, tNAA, or tCr ratios were observed between patients with suspected antibody-positive autoimmune psychosis spectrum syndromes and healthy controls.
- In the patient group, positive correlations were observed between inflammatory cerebrospinal fluid markers (white blood cell count and IgG Index) and GABA/tCr and GABA/tNAA ratios in the left caudate nucleus and right isthmus cingulate and thalamus.
- In addition, positive correlations between negative symptoms in PANSS and higher GABA/tCr ratios in the right putamen were identified.

## Limitations

- A heterogeneous patient population with different clinical manifestations/disease states and associated antibodies was analysed.
- The MEGA-LASER 3D-MRSI protocol ran unstable at our facility, which caused some measurement abortions, resulting in a smaller available data set.
- The spectral quality was poor in some voxels, as a result, several regions of interest had to be excluded from final analyses.

## Introduction

Autoimmune psychosis (AP) or autoimmune psychiatric syndromes (APS) might be interpreted as oligosymptomatic subtypes of autoimmune encephalitis (AE) (Graus *et al*., 2016; Dalmau & Graus, 2018; Dalmau *et al*., 2019; Pollak *et al*., 2020; Prüss, 2021; Li *et al*., 2024). AP/APS are associated with different antibody profiles, including well-characterised neuronal/glial antibodies (e.g., against the NMDA-R, LGI1, or MOG; Graus *et al*., 2016; Dalmau *et al*., 2019; Pollak *et al*., 2020; Prüss, 2021), novel central nervous system (CNS) antibodies (e.g., against granular cells; Endres *et al*., 2022a; Li *et al*., 2024), and systemic antibodies, such as antinuclear antibodies, particularly in cases of neuropsychiatric lupus (Jeltsch-David & Muller, 2014; Legge & Hanly, 2024). In addition to testing for CNS/rheumatic antibodies in serum and cerebrospinal fluid (CSF), diagnostic evaluation for AP/APS includes routine CSF analysis, electroencephalography (EEG), magnetic resonance imaging (MRI), and — in some cases — supplementary [^18^F] fluorodeoxyglucose positron emission tomography (FDG-PET) (Endres *et al*., 2020a; Pollak *et al*., 2020). Symptoms can be objectified and monitored psychometrically using questionnaires, external ratings, and neuropsychological test batteries that quantify cognitive functions or memory performance. Routine CSF analyses typically include white-blood cell (WBC) count, immunoglobulin G (IgG) index, oligoclonal bands (OCBs), albumin quotient (AQ), and protein concentration (Engelborghs *et al*., 2017). CSF abnormalities are found in 79 to 94% of patients with NMDA-R encephalitis (Titulaer *et al*., 2013; Dürr *et al*., 2021), but are less frequent in LGI1 encephalitis, occurring in approximately 36% of cases (Dürr *et al*., 2021). EEG alterations are non-specific but occur in 84 to 90% of patients with NMDA-R encephalitis (Titulaer *et al*., 2013; Gillinder *et al*., 2019) and in about 72% of patients with LGI1 encephalitis (van Sonderen *et al*., 2016a). MRI abnormalities are less common, occurring in 33% of NMDA-R encephalitis cases (Titulaer *et al*., 2013) and 37% of LGI1 encephalitis cases. Among LGI1 patients, two-thirds show medial temporal lobe hyperintensities (van Sonderen *et al*., 2016a; van Sonderen *et al*., 2016b). Systematic reviews and case collections of patients with suspected APS report abnormalities in the CSF in 77 to 78% of patients, in the EEG in 33 to 61%, and in the MRI in 51 to 57% (Endres *et al*., 2022b; Endres *et al*., 2022c). FDG-PET could be a valuable supplementary diagnostic tool, and whole-body FDG-PET is applied to rule out malignant tumours (Baumgartner *et al.,* 2013; Leypoldt *et al.*, 2012; Graus & Dalmau, 2016; Morbelli *et al.*, 2016; Probasco *et al.*, 2017; Bacchi *et al.,* 2018; Endres *et al.*, 2019; Hellwig *et al.*, 2019; Endres *et al.*, 2021; Bordonne *et al.*, 2021).

Magnetic resonance (MR) spectroscopy (MRS) can provide crucial in vivo insights into brain neurochemistry, enabling the measurement of glutamate and gamma-aminobutyric acid (GABA) levels (dopamine, serotonin, or noradrenaline concentrations are below the detection limit and their spectra overlap with those of more abundant metabolites). MRS also measures markers of neuronal integrity, such as total N-acetylaspartate (t-NAA; sum of N-acetylaspartate and N-acetylaspartylglutamate), and energy metabolism markers, such as total creatine (tCr; sum of creatine and phosphocreatine). tNAA and tCr are considered to be relatively constant in most constellations and are therefore often used in the denominator of reported metabolite ratios (Keshavan *et al*., 1991; Ross & Bluml, 2001; Bogner *et al*., 2014; Nakahara *et al*., 2022). Single-voxel spectroscopy focusing on a specific brain region, is the most common approach (Ross & Bluml, 2001; Tebartz van Elst *et al*., 2014; Endres *et al*., 2015a; Endres *et al*., 2017a; Maier *et al*., 2020; Maier *et al*., 2022; Nakahara *et al*., 2022; Maier *et al*., 2023). In a case report of psychotic symptoms linked to NMDA-R encephalitis, reduced glutamate levels were detected using single-voxel MRS in the hypometabolic left prefrontal cortex as shown in FDG-PET (Endres *et al*., 2015b). Another APS case with axon initial segment antibodies demonstrated significant changes in glutamate levels in the right insula before and after plasmapheresis (Endres *et al*., 2023). A systematic PubMed search for ‘(autoimmune psychosis OR autoimmune psychiatric syndrome) AND (magnetic resonance spectroscopy OR MR spectroscopy OR MRS OR magnetic resonance spectroscopic imaging OR MRSI OR chemical shift imaging OR CSI)’, performed on March 15, 2025, yielded 256 hits, none of which included case–control studies. Multivoxel MRS methods allow for the investigation of larger brain areas; this is referred to as MR spectroscopic imaging (MRSI), chemical shift imaging (CSI), or “whole-brain spectroscopy” (Bogner *et al*., 2014). Neuroinflammatory changes in the glutamatergic or GABAergic system (Crowley *et al*., 2016) could indicate electrophysiological network instability in the form of a secondary “excitation-inhibition imbalance” (Tebartz van Elst *et al*., 2014; Lee *et al*., 2017) and could trigger epileptic seizures or EEG slowing, such as intermittent rhythmic delta/theta activity (IRDA/IRTA) (Tebartz van Elst *et al*., 2011; Pollak *et al*., 2020).

***The rationale*** of this project was to investigate neurochemical MRSI signatures in patients with suspected AP spectrum syndromes compared to a healthy control (HC) group, focusing on their correlations with EEG slowing, CSF findings, and psychometric/neuropsychological test results within the patient group. The systematic literature search revealed that this was the first MRSI study to examine in vivo effects of CNS antibodies on neurochemistry in this patient group.

## Methods and participants

This study was part of a transdiagnostic project focusing on EEG slowing, approved by the local Ethics Committee of the Medical Faculty at Freiburg University Hospital, Germany (application no. EK-Freiburg: 209/18).Patients and HC were included only after providing written informed consent.

### Patient and healthy control group assessment

The patient group, consisting of adults with suspected AP spectrum syndromes, was recruited from current and former patients at the Department of Psychiatry and Psychotherapy of the Medical Center, University of Freiburg, Germany. No diagnosis was made more than 10 years before the research MRI/EEG examinations. Eligible patients were adults (age ≥18 years) who tested positive for: (1) well-characterised neuronal antibodies, (2) novel CNS antibodies, or (3) systemic antinuclear (on human embryonic kidney cells) or thyroid antibodies with distinct evidence of inflammatory brain involvement in further diagnostic work-ups (i.e., they had been diagnosed with neuropsychiatric lupus or Hashimoto encephalopathy; Jeltsch-David & Muller, 2014; Graus *et al*., 2016; Legge & Hanly, 2024). Clinically, the term “psychosis” lacks precise definition in the international diagnostic criteria for AP (cf. Pollak *et al*., 2020). From a clinical perspective, this approach is justified, as autoantibody-mediated processes often do not align with classification systems for primary mental disorders, such as paranoid schizophrenia. Other authors therefore suggested the concept of APS, which was followed here for patient inclusion (Al-Diwani *et al*., 2017; Hansen *et al*., 2020), so that also a subgroup with predominant affective spectrum syndromes was included. Cases in which neurocognitive manifestations co-occurred with schizophreniform psychosis or were the predominant clinical syndrome, were classified as “schizophreniform psychosis spectrum”. Obsessive–compulsive syndromes in combination with affective syndromes were classified as “affective spectrum syndromes” (as was one severe isolated predominant obsessive–compulsive syndrome with a previously diagnosed comorbid depressive episode). Patients were included regardless of whether they had experienced acute, chronic, and (partial) remitted disease stages. A time criterion for a subacute onset (e.g., of three months as in the Pollak criteria; Pollak *et al*., 2020) was not required for study participation. Immunotherapies could have been implemented before the MRI/EEG research measurements; all psychopharmacological therapies were recorded. As part of the diagnostic routine work-up, all patients underwent blood analyses, CSF testing, clinical EEG (analysed by the responsible physicians), and conventional MRI (analysed by senior board-certified neuroradiologists). Most patients also received a cerebral FDG-PET, which was analysed by senior board-certified nuclear medicine specialists (Endres *et al*., 2020b; Endres *et al*., 2022a). Before starting the study, we assumed that almost all patients would show clinical EEG alterations based on the data available from NMDA-R encephalitis at the time (Titulaer *et al*., 2013), but this was not the case. EEGs were additionally automatically analysed by the “avg-q” software developed in house (https://github.com/berndf/avg_q). IRDA/IRTA rates were detected with a frequency between 2 Hz and 7 Hz, and the amplitude threshold for IRDA/IRTA classification was set at >1 µV (cf. Endres *et al*., 2017b; von Zedtwitz *et al*., 2025a, 2025b). IRDA/IRTA rates for pre-hyperventilation condition and IRDA/IRTA differences (IRDA/IRTA rate after versus before hyperventilation) were reported. The serum of all patients was tested for well-characterised neuronal antibodies against intracellular antigens using an immunoblot (Ravo®, Freiburg, Germany). Both, serum and CSF, were analysed for antibodies against well-characterised neuronal cell surface antigens using a fixed cell-based assay (Euroimun®, Lübeck, Germany). In addition, routine CSF parameters were determined, including WBC counts, protein levels, AQs, IgG indices, and OCBs. Most patients were additionally tested for novel CNS antibodies in serum and CSF using a tissue-based assay on unfixed murine brain tissue (Prof. Prüss, Laboratory for Autoimmune Encephalopathies, DZNE Berlin and Department of Experimental Neurology, Charité Berlin, Germany; Kreye *et al*., 2020).

The HC group was recruited via public announcements. A lifetime diagnosis of an axis I or II disorder according to the DSM-IV (assessed with a self-report questionnaire and verified using the Structural Clinical Interview SCID-I screening and SCID-II for borderline personality disorder), recent drug use (except for episodic cannabis use) within the last six months, current or past use of psychopharmacological medication, any relevant concomitant physical illness that may affect target parameters, past brain injury (e.g., traumatic brain injury, meningitis, encephalitis, seizures/epilepsy, hydrocephalus, or space-occupying processes), systemic autoimmune diseases with potential brain involvement (e.g., lupus erythematosus) or autoimmune diseases treated with immunotherapy (e.g., steroids, azathioprine) resulted in study exclusion.

Exclusion criteria for both the patient and HC group were pregnancy or lactation, lack of legal capacity or inability to comprehend the nature, significance, and scope of the study, as well as MRI-related contraindications, such as pacemakers, claustrophobia, or intrauterine contraceptive devices.

A structured questionnaire and a test battery summarised in Supplemental Table 1 were used to collect psychometric and neuropsychological data (cf. von Zedtwitz *et al*., 2025b). Missing psychometric or neuropsychological test results did not automatically lead to exclusion, as long as the eligibility criteria were met and the diagnosis was clinically established. This flexibility was particularly useful for patients who were unable to complete the entire test battery due to their illness.

**Table 1.** Characteristics and routine diagnostic findings of the suspected autoimmune psychosis spectrum syndrome group. Only the predominant antibody was mentioned (if several antibodies were positive)

	Patients (*N* = 28)		Patients (*N* = 28)
**Antibody findings**			
**Well-characterized antibodies (*N* = 28)**	11 (39%)		
**In CSF**	4 (14%)	**In serum**	10 (36%)
Anti-LGI1 Anti-NMDA-R Anti-MOG Anti-Yo Anti-CASPR2 Anti-VGCCN Anti-TPO ANAs	1 (3.6%) 2 (7.1%) 0 (0%) 0 (0%) 0 (0%) 0 (0%) 0 (0%) 1 (3.6%)	Anti-LGI1 Anti-NMDA-R Anti-MOG Anti-Yo Anti-CASPR2 Anti-VGCCN Anti-TPO ANAs	1 (3.6%) 1 (7.1%) 2 (7.1%) 1 (3.6%) 1 (3.6%) 1 (3.6%) 2 (7.1%) 1 (3.6%)
**Novel CNS antibodies (N = 28)**	17 (61%)		
**In CSF**	16 (57%)	**In serum**	10 (36%)
Anti-vessel pattern Anti-granule cell pattern Anti-cytoplasmic pattern Anti-glia cell pattern Anti-axon initial segment pattern Anti-perinuclear pattern Anti-neuropil pattern Anti-myelin pattern Anti-Purkinje cell pattern Anti-hippocampal pattern	3 (10.7%) 4 (14.2%) 0 (0%) 1 (3.6%) 1 (3.6%) 3 (10.7%) 1 (3.6%) 1 (3.6%) 1 (3.6%) 1 (3.6%)	Anti-vessel pattern Anti-granule cell pattern Anti-cytoplasmic pattern Anti-glia cell pattern Anti-axon initial segment pattern Anti-perinuclear pattern Anti-neuropil pattern Anti-myelin pattern Anti-Purkinje cell pattern Anti-hippocampal pattern	2 (7.1%) 2 (7.1%) 1 (3.6%) 0 (0%) 1 (3.6%) 2 (7.1%) 1 (3.6%) 1 (3.6%) 0 (0%) 0 (0%)
**Clinical course**			
**Syndrome overall**		**Clinical state**	
Predominant schizophreniform psychotic syndrome Predominant affective spectrum syndrome	15 (54%)* 13 (46%)**	Acute state (Partial) remission Chronic disease ( >2 years)	7 (25%) 12 (43%) 9 (32%)
* 7 patients with additional affective syndromes, 1 patient with additional obsessive–compulsive syndrome, 1 patient with additional neurocognitive syndrome ** 3 patients with (additional) obsessive–compulsive syndromes, 1 patient with additional neurocognitive symptoms			
**Current psychopharmacological medication**		**Previous psychopharmacological medication**	
Antidepressants Typical Antipsychotics Atypical Antipsychotics Anticonvulsants Mood Stabilizers Benzodiazepines **Overall**	10 (36%) 3 (11%) 17 (61%) 3 (11%) 5 (18%) 4 (14%) **24 (88%)**	Antidepressants Typical Antipsychotics Atypical Antipsychotics Anticonvulsants Mood Stabilizers Benzodiazepines **Overall**	16 (57%) 1 (3.6 %) 14 (50%) 7 (25%) 1 (3.6%) 6 (21%) **17 (61%)**
**Diagnostic findings**			
**CSF findings (*N* = 28)**		**Visual EEG pathologies (*N* = 28)**	
Increased WBC counts Age-adjusted albumin quotient Increased protein Increased IgG index OCB in serum OCB in CSF **Overall**	3 (11%) 8 (29%) 8 (30%) (*N* = 27) 2 (7%) 0 (0%) 4 (14%) **14 (50%)**	Focal slowing Intermittent generalized slowing Continuous generalized slowing Epileptic activity **Overall**	1 (3.6%) 7 (25%) 1 (3.6 %) 0 (0%) **9 (32%)**
**Clinical routine MRI (*N* = 28)**		**FDG-PET pathologies (*N* = 18)**	
Non-specific white matter changes (Chronic) inflammatory lesions Global atrophy Focal atrophy Pineal cyst Arachnoid cyst Old defect with hemosiderin deposit Normal pressure hydrocephalus **Overall**	22 (79%) 3 (11%) 5 (18%) 3 (11%) 5 (18%) 1 (3.6%) 1 (3.6%) 1 (3.6%) **23 (82%)**	Hypermetabolism Hypometabolism **Overall**	8 (44 %) 4 (22%) **9 (50%)**

Abbreviations: ANAs, Anti-nuclear antibodies; AP, Autoimmune Psychosis; CASPR2, Contactin-associated protein-like 2; CSF, Cerebrospinal Fluid; CNS, Central Nervous System; EEG, Electroencephalography; FDG-PET, [^18^F] Fluorodeoxyglucose positron emission tomography; HC, Healthy Controls; HV, Hyperventilation; IRDA/IRTA, Intermittent Rhythmic Delta/Theta Activity, LGI1, Leucine-rich glioma-inactivated 1; MOG, myelin oligodendrocyte glycoprotein; MRI, Magnetic resonance imaging; N, Number; NMDA-R, N-methyl-D-aspartate receptor; OCBs, oligoclonal bands; TPO, Thyroperoxidase; WBC, white blood cell.

**Figure 1.**
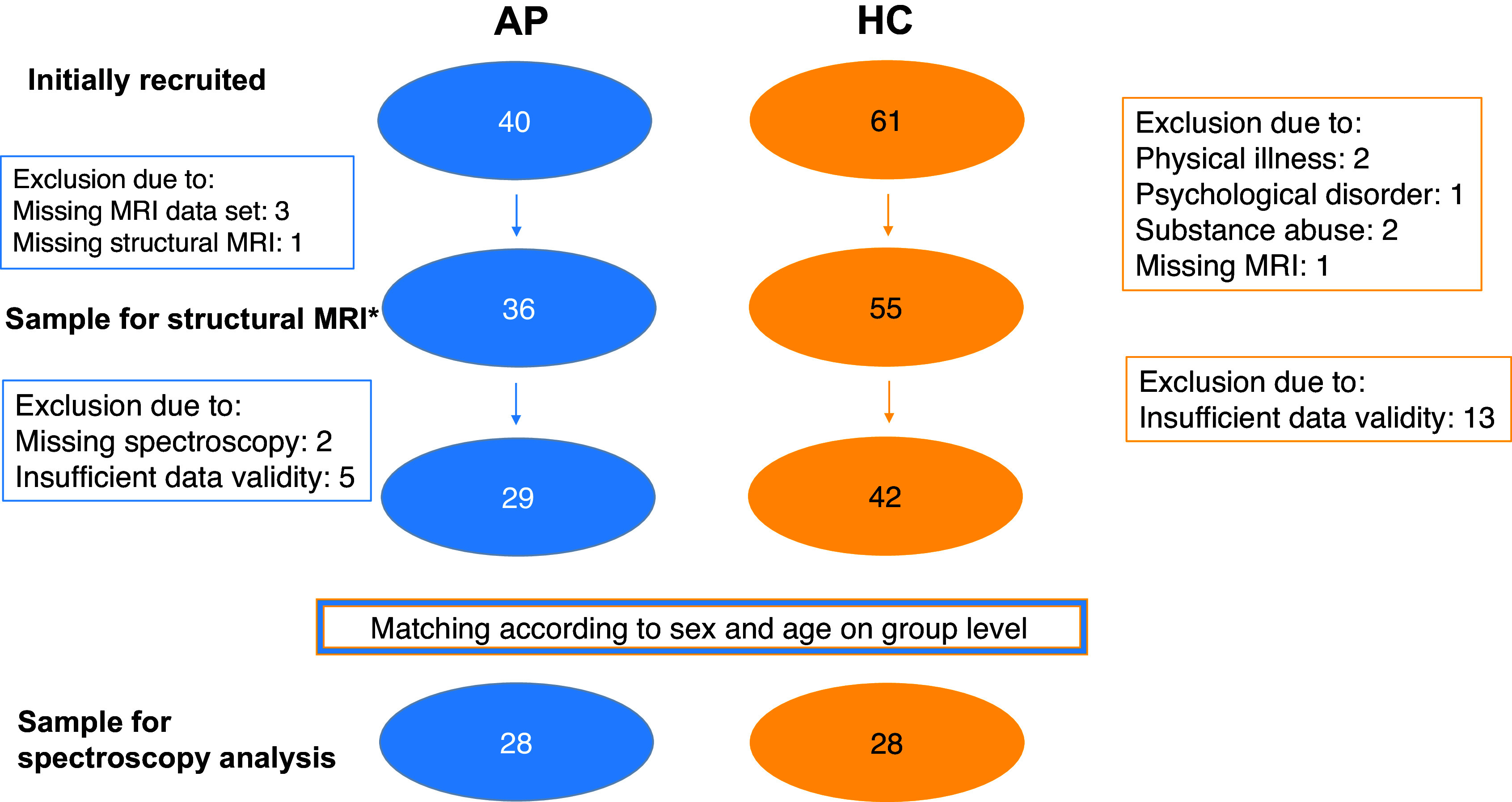
Recruitment flowchart. *The morphometric imaging data were described in another publication (von Zedtwitz *et al*., 2025b). Abbreviations: AP, autoimmune psychosis; HC, heathy controls; MRI, magnetic resonance imaging.

A total of 40 suspected AP spectrum patients and 61 HCs initially agreed to participate. The results of the structural MRI data have been published seperately (Zedtwitz *et al*., 2025b). Reasons for exclusion are summarised in Fig. 1. Following an automatic matching approach to account for significant group differences in age and sex, data from 28 patients and 28 HCs could be analysed.

### Magnetic resonance spectroscopy measurements

**Figure 2.**
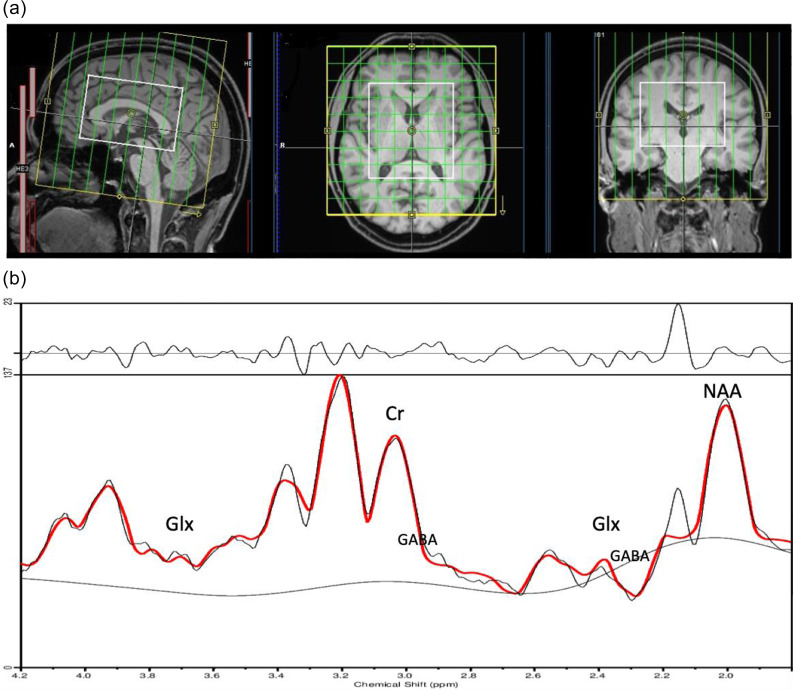
The voxels studied in magnetic resonance spectroscopic imaging are shown in A). A total of 16×16×16 voxels with a size of 1 cm^2^ were examined. The field of view area is shown in yellow. B) shows an exemplary spectrum. Abbreviations: GABA, gamma-aminobutyric acid; Glx, glutamate+glutamine; ppm, parts per million; NAA, N-acetylaspartate; Cr, creatine.

MRI and MRS measurements were performed at the imaging centre of the University Medical Center Freiburg in Germany using a Magnetom Prisma Fit 3 T system (Siemens Healthineers®, Erlangen, Germany) equipped with a 64-channel head and neck coil. High-resolution anatomical images were acquired using a T1-weighted magnetisation prepared rapid gradient echo (MPRAGE) sequence (time of repetition [TR]: 2000 ms; echo time [TE]: 4.11 ms; field of view [FOV] = 256 × 256 × 160 mm^3^; voxel size = 1 × 1 × 1 mm^3^). These images were used for region-specific spectroscopy analysis and to accurately position the MRSI volume of interest (VOI) along the anterior to the posterior commissure line. For MRSI, a spiral-encoded Mescher-Garwood localised adiabatic selective refocusing 3D-MRSI sequence (“MEGA-LASER 3D-MRSI”) (Bogner *et al*., 2014) was applied with the following parameters: TR = 1600 ms, TE = 68 ms, flip angle = 90°, and 32 averages. The MRSI protocol included real-time motion and scanner instability correction (Bogner *et al*., 2014). In addition, the LASER technique was used to reduce B1 inhomogeneity artefacts, minimise chemical shift errors, and improve the signal-to-noise ratio. The VOI was sized 80 × 90 × 80 mm and centered within a FOV of 160 × 160 × 160 mm, which encompassed the entire brain in most cases (see Fig. 2a). However, in a few instances, the cerebellum was not fully captured. The nominal voxel size was 4.096 cm^3^ with a resolution of 10×10×10 voxels. By interpolation, a voxel size of 1 cm^3^ could be achieved with 16×16×16 voxels. In edit-on/edit-off mode, the Gaussian editing pulses were applied at 1.9/7.5 parts per million (ppm) with a specified bandwidth of 60 Hz for a duration of 14.8 ms, and the centre frequency of the localisation pulses was 3.0 ppm. A total of 32 acquisition-weighted averages were acquired and a two-step phase cycle was used.

### Magnetic resonance spectroscopy analyses

**Figure 3.**
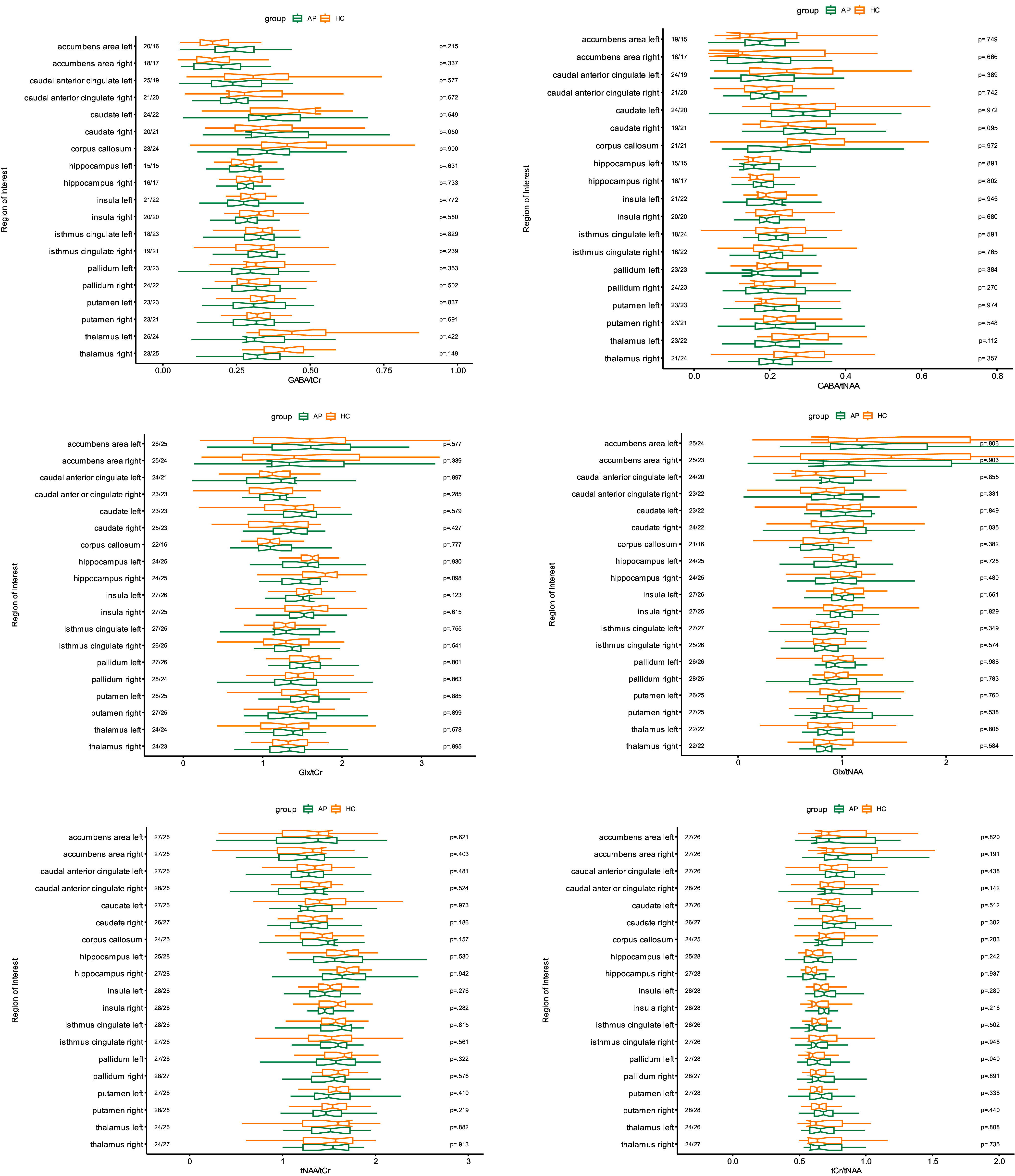
Group comparisons of magnetic resonance spectroscopic imaging-derived ratios to tCr (left) and tNAA (right) are illustrated. The data presented here are adjusted for the Cramér-Rao-Lower bounds, but not corrected for multiple testing. *Significantly higher Glx/tCr ratio in the right caudate nucleus of the suspected AP spectrum syndrome group compared to the healthy control group and a significantly lower tCr/tNAA ratio in the left pallidum were identified; however, no significant results were present after correction for multiple comparisons (padj = 0.713 and padj = 0.656, respectively). Abbreviations: AP, autoimmune psychosis; HC, healthy controls; GABA, gamma-aminobutyric acid; Glx, glutamate + glutamine; tNAA, total Nacetyl aspartate; tCr, total creatine.

The MRSI data were processed using a two-step pipeline developed by Bogner *et al*. (2014) and Spurny *et al*. (2019). This approach integrates several tools, including Matlab version 2018a (MathWorks, Natick, MA, USA), Bash (Bourne-Again Shell, GNU Operating System), MINC version 1.5 (Medical Image NetCDF, McConnel Brain Imaging Center, Montreal, QC, Canada), linear combination of model spectra (LCModel) version 6.3 by S. Provencher (Oakville, ON, Canada; Provencher, 2001), and FreeSurfer (version 6.0; Fischl, 2012). This pipeline facilitates automatic identification and quantification of the metabolite ratios from individual voxel spectra (Fig. 2b). In the first step, LCModel was applied to each voxel to estimate neurochemical concentrations based on individual voxel spectra. This involved automatic estimation of metabolite signals with the corresponding Cramér-Rao lower bounds (CRLBs), and spectral quality measures such as signal-to-noise ratio and line width of the spectrum. Voxels were classified as “valid” if they met the criterion of CRLB<30 for Glx, GABA, tCr, and tNAA ratios. In the second step, voxel-wise metabolite estimates were mapped onto FreeSurfer-derived regions of interest (ROIs) (details see Spurny *et al*., 2019). This relied on the automatic segmentation of structural brain data in FreeSurfer (version 6.0) using the recon-all command with default parameters on T1-weighted MPRAGE images to create structural ROI masks. These masks enabled regional quantification of metabolite concentrations by calculating the mean values across all valid voxels within each ROI. Regions with less than 80% voxels valid for tCr were excluded from the analysis and for the sub-analyses, regions with <80% valid voxels for the corresponding metabolites were also excluded. Additionally, participants with fewer than 80% valid voxels in more than 50% of the ROIs were excluded. To minimise susceptibility artefacts, the FOV was set slightly larger than in Spurny *et al*. (2019). Ultimately, 16 ROIs could be included in the final analysis (Fig. 3).

Group comparisons of metabolite ratios, including GABA/tNAA, GABA/tCr, Glx/tNAA, Glx/tCr, tNAA/tCr, and tCr/tNAA, were conducted for the selected ROIs. The pipeline ensured robust and region-specific quantification of neurochemical metabolites, facilitating comparisons across groups while adhering to rigorous quality control criteria.

### Statistical analyses

Statistical analyses were conducted using R software version 3.6.0 (R Foundation for Statistical Computing, Vienna, Austria). Group comparisons of clinical data for categorical variables (e.g., sex) were performed using the chi-squared (χ^2^) test, while continuous variables (e.g., age) were analysed using the Welch two sample *t*-test or the non-parametric Mann–Whitney *U* test for independent samples. For the spectroscopy data, in a first step, the normality of metabolite ratios using the Shapiro–Wilk test in R (rstatix) was evaluated and the distribution of ratios with histograms was visually inspected. These analyses indicated that the metabolite ratios did not follow a normal distribution. To identify potential covariates (sex, age, body mass index, cigarette packs per year, intelligence quotient [IQ], CRLBs of the according metabolites in the ratio) influencing the metabolite ratios, the Boruta feature selection algorithm (Boruta package) was employed on the data. Among the tested potentially confounding variables, only the CRLBs emerged as a significant covariate. To adjust for the influence of CRLBs on metabolite ratios, a robust linear model (lmRob, a robust package) was applied, to predict and remove the influence of CRLBs for the corresponding metabolite ratios, re-centering the metabolite ratios based on the mean CRLBs of the two metabolites of each ratio across all metabolite ratios and valid ROIs. All subsequent statistical analyses were performed using these adjusted ratios. Given that the MRSI results were mostly non-normally distributed, robust statistics using Wilcoxon rank sum test were used for group comparisons. For correlation analyses, robust correlations were calculated using the pbcor function (WRS2 package; Wilcox, 2012). For the test of attentional performance (TAP) results, again the Boruta model was applied to assess a potential influence of age and sex on the raw test measures. Age emerged as the only significant covariate affecting the TAP results. To adjust for age-related effects, a linear model (lm(TAP ∼ age)) and the predict function were used to re-center the test measures to the mean age, ensuring comparability across participants. Subsequent analyses were then performed using these age-adjusted TAP scores. Finally, all results were corrected for multiple comparisons using the Benjamini–Hochberg approach (Benjamini and Hochberg, 1995), with a significance threshold of *p* < 0.05. A “trend” was considered to exist when findings were significantly different before (but not after) correction for multiple comparisons.

## Results

### Study sample

The characteristics of the study sample are summarised in Tables 1 and 2. The patients with suspected AP spectrum syndromes were on average 41.8 ± 15.5 years old with 57% being female. There were no significant differences in age (*p* = 0.069) and sex (*p* > 0.999) compared to the HC group. The patient group had a significantly lower level of education (*p* = 0.005). Clinically, the patients most frequently showed predominant classical schizophreniform syndromes (54%). Only 25% of the patients were in an acute stage of the disease, while 68% had received immunotherapies prior to undergoing advanced MRI examinations. The exact clinical constellations, disease stages, and all routine diagnostic findings including different CNS antibodies are summarised in Table 1. Well-characterised antibodies were identified in 39% of patients, while 61% tested positive for novel anti-CNS antibodies. Antibody testing in the CSF was positive in 71% of all patients. As part of the routine diagnostic work-up, all patients received CSF, EEG, and MRI investigations; cerebral FDG-PET was performed in 64% of the patients. MRI alterations, including non-specific white matter changes, were identified in 82% (excluding non-specific white-matter changes or other norm variants, the MRI revealed pathological findings in only 29%), CSF abnormalities in 50%, altered cerebral FDG-PET metabolism also in 50% and clinical EEG changes in 32% of patients. While psychopharmocological medication was an exclusion criterion in the control group, 88% of the patients were taking psychopharmacological medication at the time of the study or within the preceding 6 months. The patient group achieved significantly more pathological scores in all psychometric tests as shown in Table 3, with the exception of the Wender Utah Rating Scale, the Cambridge Behaviour Scale-40 and two subtests of the Eppendorf Schizophrenia Inventory (ESI). In the neuropsychological test battery, the patient group achieved significantly lower results in verbal learning and memory tests (VLMT) for tests of learning ability, recognition and consolidation, as well as in most TAP subtests, including alertness, working memory, mental flexibility, and divided attention. The patient group showed significantly lower IQ levels in the Culture Fair Intelligence Test (CFT20R; Table 4).

**Table 2.** Group comparison of sociodemographic characteristics

Sociodemographic characteristics	Suspected AP spectrum syndromes (*N* = 28)^1^	HC (*N* = 28)^1^	*p*-value^2^
**Sex**			>0.999
Female	16 (57%); *N* = 28	15 (54%); *N* = 28	
Male	12 (43%); *N* = 28	13 (46%); *N* = 28	
**Age**	42 ± 15.5; *N* = 28	35 ± 10.5; *N* = 28	0.069
**Education level**			0.005
University degree	6 (21%); *N* = 28	18 (64%); *N* = 28	
High degree	6 (21%); *N* = 28	6 (21%); *N* = 28	
Middle degree	10 (36%); *N* = 28	4 (14%); *N* = 28	
Low degree	2 (7.1%); *N* = 28	0 (0%); *N* = 28	
Special education degree	1 (3.6%); *N* = 28	0 (0%); *N* = 28	
Other qualification	3 (11%); *N* = 28	0 (0%); *N* = 28	
**Employment status**			<0.001
Retired	10 (36%); *N* = 28	0 (0%); *N* = 28	
Unemployed	6 (21%); *N* = 28	1 (3.6%); *N* = 28	
Apprentice	1 (3.6%); *N* = 28	2 (7.1%); *N* = 28	
Student	3 (11%); *N* = 28	6 (21%); *N* = 28	
Part-time job	6 (21%); *N* = 28	7 (25%); *N* = 28	
Full-time job	2 (7.1%); *N* = 28	12 (43%); *N* = 28	
**Marital status**			0.438
Single/unwed	16 (57%); *N* = 28	20 (71%); *N* = 28	
Married	9 (32%); *N* = 28	7 (25%); *N* = 28	
Divorced	3 (11%); *N* = 28	1 (3.6%); *N* = 28	
Handedness*			0.057
Both	2 (8.3%); *N* = 24	0 (0%); *N* = 28	
Left	0 (0%); *N* = 24	4 (14%); *N* = 28	
Right	22 (92%); *N* = 24	24 (86%); *N* = 28	
**BMI**	25.4 ± 5.6; *N* = 28	22.9 ± 2.4; *N* = 28	0.031
BMI < 18	2 (7.1%); *N* = 28	0 (0%); *N* = 28	0.491
**Tabacco**	7 (25%); *N* = 28	4 (14%); *N* = 28	0.503
Package years*	2.39 ± 8.10; *N* = 27	0.27 ± 0.86; *N* = 28	0.188

* Data not available for all patients.

1 *n* (%); Mean ± SD.

2 Pearson’s Chi-squared test; Wilcoxon rank sum test; Fisher’s exact test. Abbreviations: AP, Autoimmune Psychosis; BMI, Body Mass Index; HC, Healthy Controls; SD, Standard Deviation.

**Table 3.** Group comparison in psychometry

Psychometric scores	*p*-value^1^	Psychometry	*p*-value^1^
ESI attention and speech impairment (25 AP/27 HC)	0.003	BDI II sum (26/28) STAI G state (26/28)	<0.001 <0.001
ESI auditory uncertainty (25/27)	0.037	STAI G trait (26/28)	<0.001
ESI deviant perception (28/27)	0.064	STAXI state (24/28)	<0.001
ESI ideas of reference (25/26)	0.031	STAXI trait R (24/28)	<0.001
ESI frankness (25/27)	0.441	STAXI trait T (28/28) WURS sum (23/28)	0.027 0.668
PANSS sum (26/28)	<0.001	ADHD CL sum (22/28)	0.003
PANSS positive (27/28)	<0.001	AQ sum (26/28)	<0.001
PANSS negative (27/28)	<0.001	EQ sum (26/28)	0.064
PANSS general (27/28)	<0.001		

1 Welch Two Sample t-test or Fisher’s exact test. Abbreviations: ADHD CL, ADHD-Checklist for DSM-IV; AP, Autoimmune Psychosis; AQ, Autism Spectrum Quotient; BDI II, Beck Depression Inventory II; ESI, Eppendorf Schizophrenia Inventory; EQ, Empathy Quotient; HC, Healthy Controls; PANSS, Positive and Negative Symptom Scale; STAI-G, State-Trait-Anxiety-Inventory; STAXI, State-Trait-Anger-Inventory; WURS, Wender Utah Rating Scale.

**Table 4.** Group comparisons in neuropsychological findings

Neuropsychological results	*p*-value^1^	Neuropsychological results	*p*-value^1^
**VLMT**		**TAP**	
Learning (28 AP/ 28 HC)	<0.001	Alertness no warning tone (23/27)	0.024
False positive (28/28)	0.091	Alertness warning tone (23/27)	0.062
Perservations (28/28)	0.820	Phasic alertness (23/27)	0.064
Recognition (28/28)	<0.001	Working memory mistakes (23/27)	<0.001
Consolidation (28/28)	<0.001	Working memory missings (23/27)	0.001
**CFT20R**		Mental flexibility (22/27)	0.002
IQ	<0.001	Divided attention mistakes (22/27)	0.076
		Divided attention missings (22/27)	0.006

1 Wilcoxon rank sum test. Abbreviations: AP, Autoimmune Psychosis; CFT20R, Culture Fair Intelligence Testing; HC, Healthy Controls; IQ, Intelligence Quotient; TAP, Test for Attentional Performances.

### MRSI group comparisons

The results of the MRSI across all included ROIs are summarised in Fig. 3. Before corrections for multiple testing, trends were observed, including higher Glx/tNAA ratios in the right caudate nucleus of the patient group compared to the HC group (*p* = 0.035) and lower tCr/tNAA ratios in the left pallidum (*p* = 0.040); however, these findings did not remain significant after corrections for multiple comparisons (*p*_adj_ = 0.656 and *p*_adj_ = 0.713, respectively).

### Correlation analysis within the patient group

***Electroencephalography:*** The correlation analyses of the MRSI data with the automatically analysed IRDA/IRTA rates within the patient group showed no significant associations after correction for multiple testing. Before corrections for multiple comparisons, negative correlations were observed between IRDA/IRTA rates before hyperventilation and the GABA/tCr ratio in the hippocampus bilaterally and with the GABA/tNAA ratio in the right hippocampus, as well as the Glx/tCr ratio in the right putamen and pallidum. In addition, there was a negative uncorrected correlation of the IRDA/IRTA difference with the GABA/tNAA in the right thalamus.

**Figure 4.**
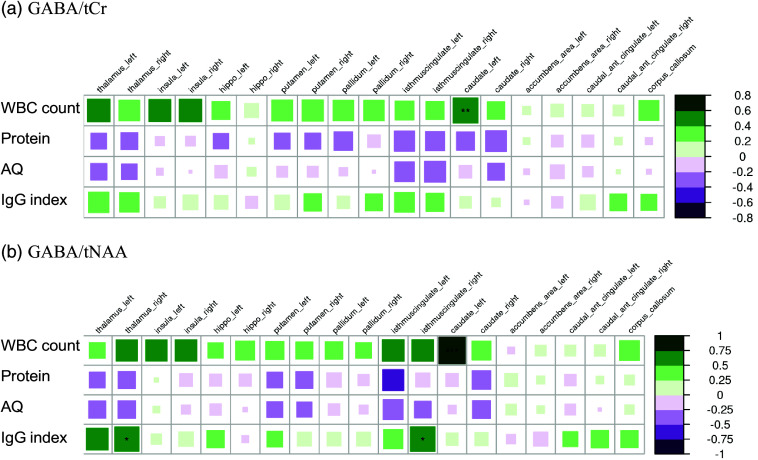
Correlations of cerebrospinal fluid data with A) GABA/tCr and B) GABA/tNAA in the suspected autoimmune psychosis spectrum syndromes. The larger and more colorful the boxes are, the closer the described correlation is to significance. If an asterisk (*/**/***) appears, significance is achieved. The number of asterisks describes the significance level as follows: *: *p*-value < 0.05; **: *p*-value<0.01; ***: *p*value< 0.001. Abbreviations: GABA, gamma-aminobutyric acid; Glx, glutamate+glutamine; IgG, immunoglobulin G; tNAA, total N-acetylaspartate; tCr, total creatine.

***Cerebrospinal fluid:*** The correlation analysis between the MRSI data and CSF findings revealed several positive correlations between WBC count and IgG Index and GABA/tCr and GABA/tNAA ratios in the left caudate nucleus (with WBC count), right isthmus cingulate and right thalamus (both with IgG index), that remained significant after correction for multiple comparisons, as indicated in Fig. 4. No significant correlations between blood brain barrier parameters (albumin quotient and protein concentration) and MRSI data were observed.

***Psychometry:*** After correction for multiple comparisons, only one significant correlation between the MRSI results and psychometric data was identified: Higher scores on the negative scale of the Positive and Negative Symptom Scale (PANSS) were associated with higher GABA/tCr ratios in the right putamen within the patient group.

***Neuropsychology:*** The correlations between the MRSI and neuropsychological data showed that higher Glx/tNAA ratios in the right hippocampus correlated with higher scores in VLMT recognition and higher GABA/tCr ratios in the left cingulate isthmus correlated negatively with divided attention mistakes in the TAP test battery. High IQ scores on the CFT20R were associated with higher tNAA/tCr ratio and lower tCr/tNAA ratio in the right caudate nucleus. All these correlations were significant after correction for multiple comparisons.

## Discussion

The main findings of this study were an absence of group differences in metabolite concentrations. Explorative analyses detected a trend for higher Glx ratios in the right caudate nucleus and an association between low IRDA/IRTA rates and high GABA ratios in different brain regions. Strong correlations between inflammatory CSF markers and GABA ratios were detected in distinct brain regions. Higher scores in the PANSS negative scale were distinctly associated with increased GABA levels in the right putamen.

***The group comparisons*** did not reveal significant differences in the measured metabolite ratios between the patient and HC group. These results could be explained by the heterogeneity of the patient collective with different antibodies (potentially targeting different brain structures), clinical presentations, and disease stages. Glutamate release, which is discussed as a general consequence of inflammatory processes in the CNS (Haroon & Miller, 2024), does not appear to be a persistent result of antibody-mediated effects. Only the trend of higher Glx ratios in the right caudate nucleus could be interpreted in this direction. In addition, no tendencies towards an altered GABA metabolism were evident. Glutamatergic and GABAergic changes could be directly explained by antibodies against glutamate (e.g., AMPA-R, NMDA-R, GluR1/5) and GABA-A/B receptors or by antibodies against the enzyme GAD65, which converts glutamate to GABA (Dalmau *et al*., 2017). Therefore, no group effects could be expected as a result of direct antibody effects, as only two patients within this study cohort had glutamatergic NMDA-R antibodies, and no patient had GABAergic antibodies. Nevertheless, these antibodies could lead to significant neurotransmitter changes when examining such patients, as we have been able to demonstrate in individual case studies analysing neurotransmitters in CSF previously (von Zedtwitz *et al*., 2025b). In addition, notably, the patient group in this study was likelier to demonstrate low-grade autoimmune-mediated neuroinflammation. This becomes evident from the additional diagnostic findings, which only revealed abnormal findings in clinical EEG in 32% and in the CSF/FDG-PET of 50%, which is rarer than in established AE cases (Titulaer *et al*., 2013; van Sonderen *et al*., 2016a; Gillinder *et al*., 2019; Dürr *et al*., 2021). Moreover, since MRS measures bulk metabolite concentrations (or ratios) and cannot discern between intra- and extracellular glutamate or GABA, this limitation could also explain the absence of observed group differences. There was a trend towards lower tCr/tNAA in the left pallidum. tCr, a marker for energy metabolism, is mostly relatively stable and is therefore usually used in the denominator of investigated metabolite ratios in MRSI research (Ross & Bluml, 2001). No changes in tNAA/tCr ratios were detected. tNAA is a marker of neuronal integrity, which is typically reduced in conditions such as hypoxia, ischaemia, neoplasm, or multiple sclerosis (Ross & Bluml, 2001) but does not seem to be relevantly changed in the suspected AP spectrum syndromes. In summary, MRS using the MRSI approach appears to have less clinical relevance to the context of diagnosing patients with suspected AP spectrum syndromes, but raises hopes of being able to contribute to the understanding of pathophysiological processes in the context of a multimodal diagnostic assessment.

***A nominal correlation between EEG slowing before hyperventilation and GABA ratios*** in the hippocampus, an association between IRDA/IRTA difference with the GABA/tNAA ratio in the right thalamus, as well as a correlation between EEG slowing and Glx ratios in the right putamen and pallidum were observed. These findings might indicate that changes in GABA, the primary inhibitory neurotransmitter, and Glx, the principal excitatory neurotransmitter, are associated with increased EEG slowing. Such findings would be consistent with an underlying excitation-inhibition imbalance in some patients (Tebartz van Elst *et al*., 2014; Lee *et al*., 2017) and might at least partly explain the clinically observed EEG alterations in this patient group and align with previously published AP/APS cohorts (Endres *et al*., 2022b; Endres *et al*., 2022c). However, these findings stem from explorative analyses and were not significant after corrections for multiple testing; thus, these interpretations should be taken with caution and cannot be deduced unequivocally from the findings of this study.

***Inflammatory CSF markers and GABA ratios*** showed a significant positive correlation in the left caudate nucleus, right isthmus cingulate, and right thalamus. They indicate alterations in the GABAergic system in the context of neuroinflammatory markers (i.e., WBC count and IgG index), which are typically elevated in acute/chronic neuroinflammatory constellations (Graus *et al*., 2016; Engelborghs *et al*., 2017; Pollak *et al*., 2020). These findings may provide evidence for an excitation-inhibition imbalance caused by inflammatory processes (Lee *et al*., 2017; Tebartz van Elst *et al*., 2014), which would also be supported by the trend for correlations between the GABA and Glx ratios and the IRDA/IRTA rates (Tebartz van Elst *et al*., 2011).

***Correlations with psychometric and neuropsychological scores*** revealed one psychometric and several neuropsychological associations with neurometabolites. Higher GABA/tCr ratios in the right putamen correlating with PANSS negative symptoms could provide a pathophysiological basis for aberrant frontostriatal connectivity in suspected AP spectrum patients, which has previously been associated with negative symptoms in patients with primary schizophrenia (Shukla *et al*., 2019). Neuropsychological correlations were observed depending on the test in different regions with different neurometabolites. These findings suggest that MRSI is possibly less capable to depict disease-specific processes, but rather superordinate neuropsychological alterations. Similar findings have also been made in neurology, where MRSI is used to detect brain tumours, but no specific tumour subentity can be diagnosed with it (Ross & Bluml, 2001).

***Several limitations*** of this study must be addressed. In contrast to the morphometric analysis of the study cohort, which comprised 35 patients (von Zedtwitz *et al*., 2025b), only 28 patients were included in this analysis. This was due to the instability of the MEGA-LASER 3D-MRSI protocol at our facility, which caused frequent failures in the MR system and, consequently, measurement abortions, resulting in a smaller available dataset. In addition, the spectral quality was poor in some voxels; consequently, numerous ROIs had to be excluded (see the number of cases in Fig. 3). However, in contrast to the single-voxel method, a relatively large volume of the brain with precise regional parcellation could be analysed (Fig. 2). Only voxels with sufficient spectral quality (based on CRLBs) of voxels/ROIs were included in the analysis. Overall, not all regions could be validly measured in all patients using the MRSI approach, and the MRS methodology, including post-processing, was complex, which limits its applicability in routine clinical practice. The study population also imposed relevant limitations. The negative findings could be explained by the heterogeneous patient population with different antibodies, clinical manifestations, and disease states. Due to the rarity of the disorder, assembling large patient groups with similar clinical syndromes, disease states, and antibodies is challenging. No such studies are currently available. Multicentre studies with homogenous patient groups (preferably with identical antibodies and same clinical syndromes) measured on the same MRI scanner would be desirable. This first case–control study provides initial pilot data upon which future studies can be based on.

## Conclusions

An advanced diagnostic approach, as used here, allows for disentangling the association of neuroinflammation (e.g., increased WBC count or IgG index), regional neurotransmitter activity (e.g., Glx or GABA ratios), and electrophysiological processes (e.g., IRDA/IRDA rates) in patients with suspected AP spectrum syndromes. Such multimodal analyses could provide a better understanding of pathophysiological processes on different levels in immunopsychiatric disorders in the future.

**Figure 5.**
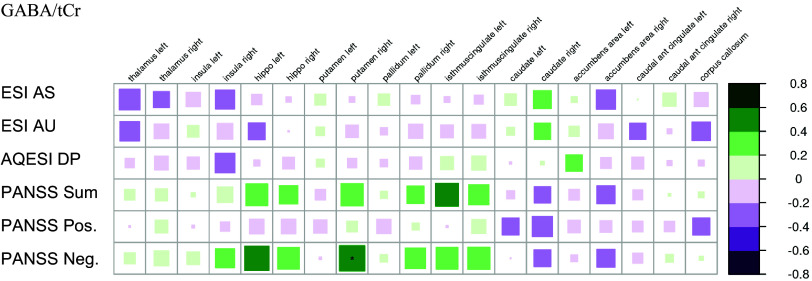
Correlations of psychometric data with GABA/tCr in the suspected autoimmune psychosis spectrum syndromes. The larger and more colorful the boxes are, the closer the described correlation is to significance. If an asterisk (*/**/***) appears, significance is achieved. The number of asterisks describes the significance level as follows: *: *p*-value < 0.05; **: *p*-value < 0.01; ***: *p*-value < 0.001. Abbreviations: ESI, Eppendorf Schizophrenia Inventory; GABA, gamma-aminobutyric acid; PANSS, positive and negative syndrome scale; tCr, total creatine.

**Figure 6.**
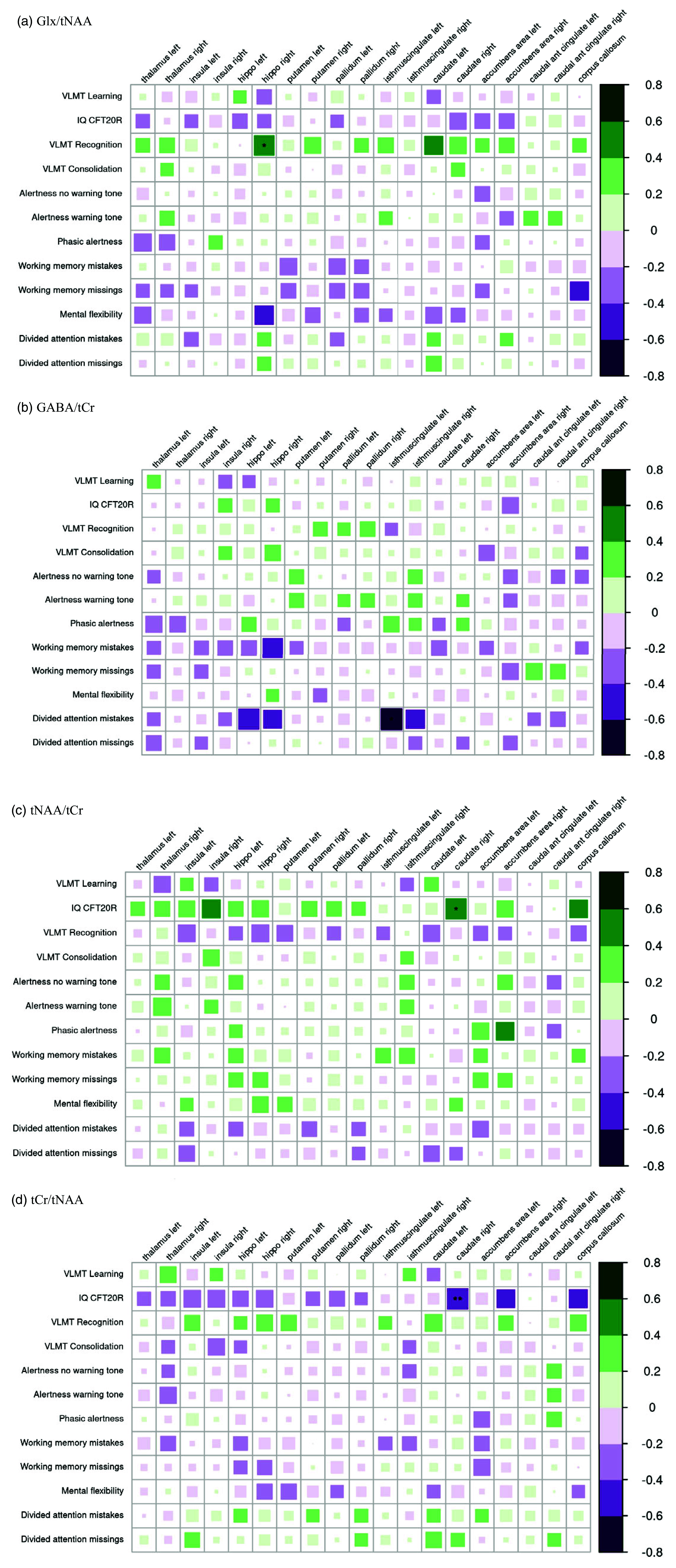
Correlations of neuropsychological data with A) Glx/tNAA, B) GABA/tCr, C) tNAA/tCr, and D) tCr/tNAA in the suspected autoimmune psychosis spectrum syndromes. The larger and more colorful the boxes are, the closer the described correlation is to significance. If an asterisk (*/**/***) appears, significance is achieved. The number of asterisks describes the significance level as follows: *: *p*-value < 0.05; **: *p*-value < 0.01; ***: *p*-value < 0.001. Abbreviations: CFT20R, Culture Fair Intelligence Testing; Glx, glutamate+glutamine; IQ, intelligence quotient; tCr, total creatine; tNAA, total N-acetylaspartate; VLMT, verbal learning and memory test.

## Supporting information

Endres et al. supplementary material Endres et al. supplementary material

## Data Availability

All necessary data can be found in the paper.

## Supplementary material

The supplementary material for this article can be found at https://doi.org/10.1017/neu.2025.10036.
