# Supplementary material for: MR spectroscopic imaging and its association with EEG, CSF, and psychometric/neuropsychological findings in patients with suspected autoimmune psychosis spectrum syndromes
Source: Acta Neuropsychiatr. 2025 Sep 17;37:e88. doi: 10.1017/neu.2025.10036 (PMC13130269; doi:10.1017/neu.2025.10036)
Supplement: Endres et al. supplementary material [file S0924270825100367sup001.docx]

**Supplementary Table**

| **Questionnaire** | **Reference** |
| --- | --- |
| Structured Clinical Interview for DSM-IV (SCID-I/SCID-II) | First et al., 1997 |
| Eppendorf Schizophrenia Inventory (ESI) | Mass et al., 2000 |
| Beck Depression Inventory II (BDI-II) | Hautzinger, 1994; Beck et al., 1961 |
| State-Trait-Anxiety-Inventory (STAI-G) | Spielberger et al., 1983 |
| State-Trait-Anger-Inventory (STAXI) | Spielberger et al., 1999 |
| Wender Utah Rating Scale (WURS) | Retz-Junginger et al. 2002 |
| ADHD-Checklist for DSM-IV (ADHD-CL) | Rösler et al. 2004 |
| Autism Spectrum Quotient (AQ) | Baron-Cohen et al., 2001 |
| Cambridge Behaviour Scale-40 Empathy quotient (EQ) | Baron-Cohen & Wheelwright, 2004 |
| Positive and Negative Syndrome Scale (PANSS) | Kay et al., 1987 |
| Test for Attentional Performances (TAP) | Zimmermann and Fimm, 2002 |
| Verbal Learning and Memory Test (VLMT) | Helmstaedter and Durwen, 1990 |
| Culture Fair Intelligence Testing (CFT-20 R) | Weiß, 2006 |

**Supplementary Table 1: Psychometric and neuropsychological test battery.** Abbreviations: ADHD, Attention deficit hyperactivity disorder. ADHD CL, ADHD-Checklist for DSM-IV; AQ, Asperger Questionnaire; BDI II, Beck Depression Inventory II; CFT-20 R, Culture Fair Intelligence Testing; ESI, Eppendorf Schizophrenia Inventory; EQ, Cambridge Behaviour Scale-40; PANSS, Positive and Negative Syndrome Scale; SCID-I/SCID-II, Structured Clinical Interview for DSM-IV; STAI-G, State-Trait-Anxiety-Inventory; STAXI, State-Trait-Anger-Inventory; TAP, Test for Attentional Performances; VLMT, Verbal Learning and Memory Test; WURS, Wender Utah Rating Scale.

**References:**

Baron-Cohen S, Wheelwright S (2004): The empathy quotient: an investigation of adults with Asperger syndrome or high functioning autism, and normal sex differences. J. Autism Dev. Disord. 34, 163–175. https://doi.org/10.1023/b:jadd.0000022607.19833.00

Baron-Cohen S, Wheelwright S, Skinner R, Martin J, Clubley E. (2001): The autism-spectrum quotient (AQ): evidence from Asperger syndrome/high-functioning autism, males and females, scientists and mathematicians. J. Autism Dev. Disord. 31, 5–17. https://doi.org/10.1023/a:1005653411471

Beck AT, Ward CH, Mendelson M, Mock J, Erbaugh J (1961): An inventory for measuring depression. Arch. Gen. Psychiatry 4, 561–571. https://doi.org/10.1001/archpsyc.1961.01710120031004

First MB, Gibbon M, Robert L, Spitzer W, Janet, BW, Benjamin LS (1997): SCID-II: Structured Clinical Interview for DSM-IV AXis II Personality Disorders: User's Guide. American Psychiatric Press.

Hautzinger M (1994): "Beck-Depressions-Inventar (BDI)." Bern: Huber.

Helmstaedter C, Durwen HF (1990): VLMT: Verbaler Lern- und Merkfähigkeitstest: Ein praktikables und differenziertes Instrumentarium zur Prüfung der verbalen Gedächtnisleistungen. [VLMT: A useful tool to assess and differentiate verbal memory performance.]. Schweiz. Arch. Für Neurol. Neurochir. Psychiatr. 141, 21–30.

Kay SR, Fiszbein A, Opler LA (1987): The positive and negative syndrome scale (PANSS) for schizophrenia. Schizophr. Bull. 13, 261–276. https://doi.org/10.1093/schbul/13.2.261

Mass R, Haasen C, Wolf K (2000): [The Eppendorf Schizophrenia Inventory (ESI). Development and evaluation of a questionnaire for assessment of characteristic self-perception of cognitive dysfunctions by schizophrenic patients]. Nervenarzt 71, 885–892. https://doi.org/10.1007/s001150050679

Retz-Junginger P, Retz W, Blocher D, Weijers HG, Trott GE, Wender PH, Rössler M (2002): [Wender Utah rating scale. The short-version for the assessment of the attention-deficit hyperactivity disorder in adults]. Nervenarzt 73, 830–838. https://doi.org/10.1007/s00115-001-1215-x

Rösler M, Retz W, Retz-Junginger P, Thome J, Supprian T, Nissen T, Stieglitz RD, Blocher D, Hengesch G, Trott GE (2004): [Tools for the diagnosis of attention-deficit/hyperactivity disorder in adults. Self-rating behaviour questionnaire and diagnostic checklist]. Nervenarzt 75, 888–895. https://doi.org/10.1007/s00115-003-1622-2

Spielberger CD (1999): State-Trait Anger Expression Inventory-2: STAXI-2 professional manual Odessa, FL: Psychological Assessment Resources

Spielberger C, Gorsuch R, Lushene R, Vagg PR, Jacobs G (1983): Manual for the State-Trait Anxiety Inventory (Form Y1 – Y2). Palo Alto, CA: Consulting Psychologists Press.

Weiß RH (2006): CFT 20-R Grundintelligenztest Skala 2-Revision. Gottingen: Hogrefe.

Zimmermann P, Fimm B (2002): Testbatterie zur Aufmerksamkeitsprüfung. PSYTEST.
